# Nursing Honeybee Behavior and Sensorial-Related Genes Are Altered by Deformed Wing Virus Variant A

**DOI:** 10.3390/insects15020080

**Published:** 2024-01-23

**Authors:** Silva Diego, Arismendi Nolberto, Alveal Juan Pablo, Ceballos Ricardo, Zapata Nelson, Vargas Marisol

**Affiliations:** 1 Laboratorios de Virología y Patologías en Abejas, Facultad de Agronomía, Universidad de Concepción, Av. Vicente Méndez 595, Chillán 3780000, Chile; diegosilva@udec.cl; 2 Centro de Investigación Austral Biotech, Facultad de Ciencias, Universidad Santo Tomás, Av. Picarte 1130–1160, Valdivia 5090000, Chile; narismendi@santotomas.cl; 3 Laboratorio de Ecología Química, Instituto de Investigaciones Agropecuarias, INIA Quilamapu, Av. Vicente Méndez 515, Chillán 3780000, Chile; juanpauloalveal@gmail.com (A.J.P.); rceballos@inia.cl (C.R.); 4 Laboratorio de Fitoquímica, Facultad de Agronomía, Universidad de Concepción, Av. Vicente Méndez 595, Chillán 3780000, Chile; nzapata@udec.cl

**Keywords:** nurse bees, DWV-A, OBPs, synaptic genes, olfactory responses

## Abstract

**Simple Summary:**

Honeybees, *Apis melllifera*, are the most widely used bees in the world for pollination services. However, in recent years, continuous colony losses have been recorded worldwide. One of the factors behind these losses is associated with diseases specific to this species, such as those caused by viruses. One of the known viruses affecting honeybee populations is the deformed wing virus (DWV). DWV causes physical malformations, decreased olfactory sensitivity, learning difficulties, and behavioral alterations, which can compromise hive behaviors. Thus, we evaluated behavioral response, the expression of antenna-specific odorant-binding protein (OBP) genes, and brain genes related to bee behavior, especially nurse bees, in honeybees inoculated with the DWV variant A. We performed olfactory sensitivity analyses in beehives. We performed behavioral assays with the larvae-emitted alarm pheromone component to stimulate infected nurse bees. We found that high levels of viral replication in both the head and antennae altered the behavioral response, decreasing attraction to the pheromone component, and DWV-A infection decreased the gene expression of OBPs and brain genes. Thus, DWV-A infection in adult bees could compromise internal hive cohesion and *A. mellifera* nurse bee behaviors.

**Abstract:**

Insect behavior is coordinated mainly by smell through the diverse odor-binding proteins (OBP) that allow them to identify and recognize their environment. Sensory information collected through smell is then analyzed and interpreted in the brain, allowing for correct insect functioning. The behavior of honeybees (*Apis mellifera* L.) can be affected by different pathogens, such as deformed wing virus (DWV). In particular, the DWV variant A (DWV-A) is capable of altering olfactory sensitivity and reducing the gene expression of different OBPs, including those associated with nursing behavior. The DWV is also capable of replicating itself in the sensory lobes of the brain, further compromising the processing of sensory information. This study evaluated the behavioral response of nurse honeybees exposed to a pheromone compound and the alterations in the gene expression of the pre- and post-synaptic neuronal genes neuroxins-1 and neurogilin-1 in the bee heads and OBP proteins in the antennae of nurse bees inoculated with DWV-A. The behavioral response of nurse bees exposed to the larval pheromone compound benzyl alcohol was analyzed using a Y-tube olfactometer. The viral load, the gene expression of OBP5 and OBP11 in antennae, and neuroxins-1 and neurogilin-1 in the bee heads were analyzed via qPCR. High viral loads significantly reduced the ability of 10- and 15-day-old nurse honeybees to choose the correct pheromone compound. Also, the gene expression of OBP5, OBP11, neuroxin-1, and neurogilin-1 in nurse honeybees decreased when they were highly infected with DWV-A. These results suggest that a DWV-A infection can disturb information processing and cause nursing honeybees to reduce their activity inside the hive, altering internal cohesion.

## 1. Introduction

Bees, particularly *Apis mellifera* L., have an excellent olfactory system, which modulates highly cooperative social behavior in and outside the hive, such as brood care, foraging, hive maintenance, and cleaning, among other activities. This peripheral sensory system is located mainly in the antennae and is key to the chemosensory detection of pheromones and environmental odorants [1,2]. The sensitivity and specificity of the olfactory system are greatly attributed to the presence of odorant-binding proteins (OBPs) [3]. These OBPs are small water-soluble molecules located in the sensory dendrite of the antennae that selectively bind to different environmental compounds [4,5]. The number and diversity of OBPs vary among insect species; in *A. mellifera*, a total of 21 OBPs have been described, of which only nine are specifically expressed in the antennae [6]. Among these proteins, OBP5 and OBP11 are particularly related to the larval care process inside beehives [7,8]. These OBPs have an affinity for the volatile compounds of the warning pheromone generated via diseased larvae [7,8]. Some of the specific compounds emitted and perceived by these proteins are phenethyl acetate, phenyl ethanol, and benzyl alcohol [9]. Of these three compounds, benzyl alcohol is the most competitive compound for the OBP5 protein recognition site [7].

The decline in honeybees and other bee populations has been reported throughout the last decade [10,11], partially associated with diseases and pathogens specific to these species [12]. Viral pathogens are of great relevance because they can remain in the hives without generating detectable clinical symptoms, making their recognition difficult [13,14]. Also, viral diseases in conjunction with different stress factors, such as food quality and availability, climatic conditions. and other pathogens, such as varroa destructor Anderson and Trueman, bacteria, or fungi, can greatly contribute to the loss of bee hives [10,13,14]. One of the most important viral diseases is the deformed wing virus (DWV), which characteristically causes wing deformities in infected adult bees [15]. The DWV has also been proven to cause early mortality in infected bees [15], as well as a long-term increase in viral titers, which can generate alterations in olfactory sensitivity and foraging tasks in infected workers [16].

Three epidemiologically important variants of DWV have been described as follows: DWV-A, DWV-B, and DWV-C [17,18]. DWV-A has proven to be the predominant variant in Chile [19,20] and is able to replicate itself in the basal regions of the antennal epithelium [21]. It has also been demonstrated that the presence of this pathogen in the antennae of *A. mellifera* results in a decrease in olfactory sensitivity to different scents of plant species, as well as inducing a decrease in gene expression, especially of OBP5 and OBP11 [22]. In addition, DWV can alter the memory of *A. mellifera* because this pathogen is capable of replicating itself in brain regions, including the neuropils responsible for vision and smell [23]. Co-infestation with the varroa mite has been proven to generate the gene depletion of the pre- and post-synaptic proteins neurexin (AmNrx-1) and neuroligin (AmNlg-1), respectively, decreasing information processing and associative memory [24]. Nevertheless, the downregulation of these genes has only been observed in their interaction with varroa mites; it has not yet been determined whether the virus alone is capable of altering their expression. Therefore, the aim of this study was to evaluate the behavioral response of nurse honeybees exposed to pheromone compounds related to larvae care and also to evaluate the alterations in the gene expressions of the pre- and post-synaptic neuronal genes neuroxins-1 and neurogilin-1 in bee heads and antennae. OBP proteins in the antennae of nurse bees inoculated with DWV-A were also assessed. The results of this study aid in understanding the effects of DWV-A infections at the individual and social levels within the colony. 

## 2. Materials and Methods

### 2.1. Inoculum Preparation

The deformed wing virus variant A (DWV-A) inoculum was obtained from infected colonies following the methodology described by Gusachenko et al. [25]. Briefly, a pool of 20 honeybees that were symptomatic (deformed wings) was collected from free-flying hives from an experimental apiary located at the experimental center “El Nogal” (36°35′58.25″ S–72°04′51.77″ W), Universidad de Concepción, Chillán, Chile. Using qPCR, the following pathogens, *Nosema ceranae*, *N. apis*, *Lotmaria passim*, ABPV, CBPV, DWV-B, and DWV-A, were identified and quantified. After the molecular analysis, hives with high viral titers of DWV-A, which were free of *N. ceranae*, *N. apis*, *Lotmaria passim*, ABPV, CBPV, DWV-B were selected The bees were introduced into a stomacher bag, and 20 mL of phosphate-buffered saline (1× PBS) was added; then, for the 90s at high speed, they were homogenized in a stomacher 80 (Seward, London, UK). Subsequently, they were centrifuged, first at 1500× *g* for 10 min, followed by 10,000× *g* for 10 min, both at 4 °C. The supernatant was collected and then purified via filtration with a 0.22 µm filter (PES, Merck Millipore, Darmstadt, Germany). Finally, 200 µL of RNA was collected, and the cDNA synthesis and quantification of the viral load of the inoculum via qPCR was performed. Subsequently, the inoculum was used to inoculate newly emerged bees free of pathogens and with low viral loads. After 12–15 days post inoculation (dpi), a second viral inoculum was obtained (as described previously), and the purity of the inoculum was confirmed using qPCR (high viral load of DWV-A and free of other pathogens). Finally, it was stored at −80 °C to be used in subsequent bioassays.

### 2.2. Honeybee Inoculation

Adult *A. mellifera* specimens for the trial were supplied by the experimental apiary located at the experimental center “El Nogal” (36°35′58.25″ S–72°04′51.77″ W), Universidad de Concepción, Chillán, Chile. Brood frames were maintained under controlled conditions at 30 °C ± 1; 60% ± 3 RH. Subsequently, newly emerged bees were carefully collected and randomly confined in plastic cages (base = 8 cm diameter, mouth = 10 cm diameter, and height = 15 cm).

The experimental procedure was then carried out in a similar way to that previously published by Silva et al. [22]. In brief, one group of young bees was individually and orally inoculated (I-DWV treatment) with 5 µL of a viral suspension (1.0 × 10^9^ copy number per bee) in a 60% sucrose solution. The bees that did not consume the total viral inoculum suspension were discarded from the experiment. Another group of bees was not inoculated with the viral suspension (N-DWV treatment), which was referred to as a control group. Then, 700 worker bees were maintained in ten plastic cages, with 70 bees per cage (each individual was considered an independent replicate for future experiments), and the distribution was as follows: 350 bees infected with the viral suspension and 350 non-inoculated bees and a total of 5 cages were used for each treatment. These bees were supplemented with 3 g of a pollen replacer (commercial preparation included soybean, brewer’s yeast, corn starch, wheat flour, curbicular pollen, canola oil, and canola oil) and 60% sucrose syrup ad libitum [26].

### 2.3. Stimulus Preparation

For the behavioral assay, a pure standard of benzyl alcohol (≥99% GC, Sigma-Aldrich, Munich, Germany) and *Mentha piperita* essential oil were used as the stimuli, considering the previous results reported by Silva et al. [22]. In summary, *M. piperita* leaves were collected from ornamental specimens in Chillán, Ñuble Region, Chile. Then, 300 g of this material was air-dried at room temperature for 48 h, after which was then boiled for 6 h via hydrodistillation using a Clevenger apparatus. Finally, anhydrous sodium sulfate (Na_2_SO_4_) was added to remove water from the samples, and they were stored at 8 °C in complete darkness. Each stimulus was diluted in ethanol (≥99% GC, Sigma-Aldrich, Munich, Germany) at a concentration of 100 µL/mL [27].

### 2.4. Olfactometric Bioassays

The behavioral response of *A. mellifera* to volatiles of *M. piperita* essential oil and benzyl alcohol was conducted using a glass Y-tube olfactometer (21 cm long with an internal diameter of 3 cm) (Universidad de Concepción, Concepción, Chile), with glass odor chambers (15 cm long with an internal diameter of 3 cm) that were connected to the end of the Y-tube arms to deliver the stimulus using filtered air at 280 mL min^−1^ with a positive pressure air pump. A volume of 10 μL for each stimulus diluted in ethanol at a concentration of 100 µL/mL [27] was applied to a strip of filter paper (1 cm × 7.5 cm); 30 s was allowed for the solvents to evaporate, and then these were placed inside an odor chamber. Thirty worker honeybees that were 5-, 10-, 15-, and 20 days old (120 worker honeybees in total) were used per trial and health condition (I-DWV or N-DWV) (*n* = 320). Each worker honeybee was allowed to move freely inside the olfactometer for 6 min. The first choice made by each insect in that time frame to either arm of the Y-tube was considered a choice. The distribution of choice was defined as follows: a positive choice to the arm where the stimulus was applied, a negative choice to the arm without stimulus, and no choice when, at the end of the evaluation time, the insect did not choose the previously mentioned zones. The worker honeybees were subjected to choice tests between benzyl alcohol vs. air and essential oils vs. benzyl alcohol (total *n* = 640). After each bee had been chosen, they were stored at −80 °C until further analysis.

### 2.5. RNA Extraction and cDNA Synthesis

For RNA extraction, 13 adult bees were randomly collected from each treatment (I-DWV and N-DWV) and olfactometric assay (pheromone vs. air; pheromone vs. essential oil). The antennae and head of these previously selected bees were used for RNA extraction, according to Kim et al. [21] and Mondet et al. [28], with some modifications. Briefly, the antennae and heads were cooled to −80 °C, then triturated by adding 200 µL of TrizolTM (Invitrogen, Thermo Fisher Scientific, Waltham, MA, USA) into an Eppendrof tube (1.7 mL) using a pestle and mortar. Then, 5 µL of carrier RNA (Invitrogen, Thermo Fisher Scientific, Waltham, MA, USA), 100 µL of absolute ethanol (100%), and 200 µL of chloroform were added to the homogenate and incubated at 4 °C for 10 min. Finally, it was centrifuged for 5 min at 10,000× *g* at 4 °C, and the supernatant was collected for RNA extraction from the antennae and head, which was performed following the instructions provided by E.Z.N.A. Total RNA Kit I (Omega Bio-Tek, Norcross, GA, USA). RNA quality and yield were determined with a spectrometer (Infinite 200 PRO NanoQuant, Tecan Group, Männedorf, Switzerland). The extracted RNA was subsequently used for cDNA first-strand synthesis using an M-MLV reverse transcriptase enzyme (Invitrogen, Life Technologies, Carlsbad, CA, USA), according to the manufacturer’s instructions. The cDNA samples were stored at −20 °C for later use.

### 2.6. Real-Time PCR Quantification for Viral Load and Gene Expression

The quantification of the viral load (DWV-A) and expression of the neuronal genes, such as AmNrX-1 and AmNlG-1 [24], as well as Amobp5 (OBP5) and Amobp11 (OBP11) [2] was carried out using specific primers (Table 1). The qPCR reaction was performed according to Riveros et al. [19]. Briefly, a 1× KAPA SYBR FAST Universal 2× qPCR Master Mix (Kapa Biosystems, Wilmington, MA, USA) was used, following the supplier’s instructions for the qPCR reaction. A reaction volume of 15 µL was used, with 20 ng of cDNA, 530 nM of each primer, and sterile-filtered molecular grade water until 15 µL was reached. The reaction thermal conditions were 95 °C for 3 min, followed by 40 cycles at 95 °C for 5 s, 60 °C for 15 s and 72 °C for 15 s. Real-time PCR assays were performed on an Agilent AriaMx Real-Time PCR System (Agilent Technologies, Santa Clara, CA, USA), and data were analyzed using Agilent AriaMx 1.5 software (Stratagene, Agilent Technologies, Santa Clara, CA, USA). The relative expression of each gene was calculated after normalization with an endogenous gene (β-actin) (Table 1), as described by Pfaffl [29]. For the quantification of the viral load in each segment of the worker bees (head and antennae), a standard curve using a purified PCR product (Wizard^®^VR SV gel and PCR clean-up system, Promega, Madison, WI, USA) was used. Then, the purified amplicon was quantified via spectrophotometry (EpochTM Microplate Spectrophotometer, BioTek, Winooski, VT, USA) to calculate the copy number according to Wu et al. [30]. Linear standard curves (95–100% efficiency) were generated using serial dilutions (1.0 × 10^1^ to 1.0 × 10^9^) of the purified cDNA viral copy number. Then, Ct values were plotted against the copy number values (log10). Therefore, the sample copy number was estimated using the Ct values and comparisons with the linear equation of the standard curve and the β-actin gene clearance normalization values [31]. Finally, data were expressed as the DWV-A copy number per bee, taking into account the dilutions that were performed in the cDNA synthesis and qPCR reaction.

### 2.7. Data Analysis

Considering that the viral load quantified in the antennae and head was correlated in both treatments (DWV-A inoculated and non-inoculated bees), a nested multivariate ANOVA (MANOVA) was designed using a general linear model (GLM). Thus, viral load data quantified in the antennae and the head were considered response variables. The bee age (5-, 10-, 15- and 20 days old) and viral status (bees inoculated (I-DWV) and non-inoculated (N-DWV) with DWV-A) were considered independent variables, with the viral condition nested in the bee age variable. The Bonferroni test (*p* < 0.05) was then run to separate the means between treatments. A chi-square test (*p* < 0.05) was used to analyze the statistical difference in the responses (preference) of inoculated (I-DWV) and non-inoculated (N-DWV) honeybees at 5-, 10-, 15- and 20 days old (preference) in the olfactometric Y-tube test with benzyl alcohol and *M. piperita* essential oil. Also, a binary (1 and 0) logistic regression was run to determine the likelihood of the preference of young bees for the pheromone compound in the Y-tube test as a function of the DWV-A load and bee age. The preference for the pheromone compound was marked as positive (yes = 1), and no preference or preference for the other compound was considered a negative response (no = 0). The data on all bees tested in the experiments (including the viral load of the antenna, head, and abdomen of inoculated and non-inoculated bees) were included in the logistic regression. Statistical differences in the gene expressions of AmNrX-1, AmNlG-1, Amelobp5 (OBP5), and Amelobp11 (OBP11) were also estimated using nested MANOVA because Amelobp5 and Amelobp11, as well as AmNrX-1 and AmNlG-1 expression values, were correlated. Thus, relative gene expressions were considered response variables in the function of the independent variables, such as bee age and viral status (nested variable). Then, a Bonferroni test (*p* < 0.05) was run to separate the means between treatments. Data analysis was performed with STATISTICA 7.0 software (StatSoft, Tulsa, OK, USA).

## 3. Results

### 3.1. Viral Load in the Antenna and Head

**Figure 1:**
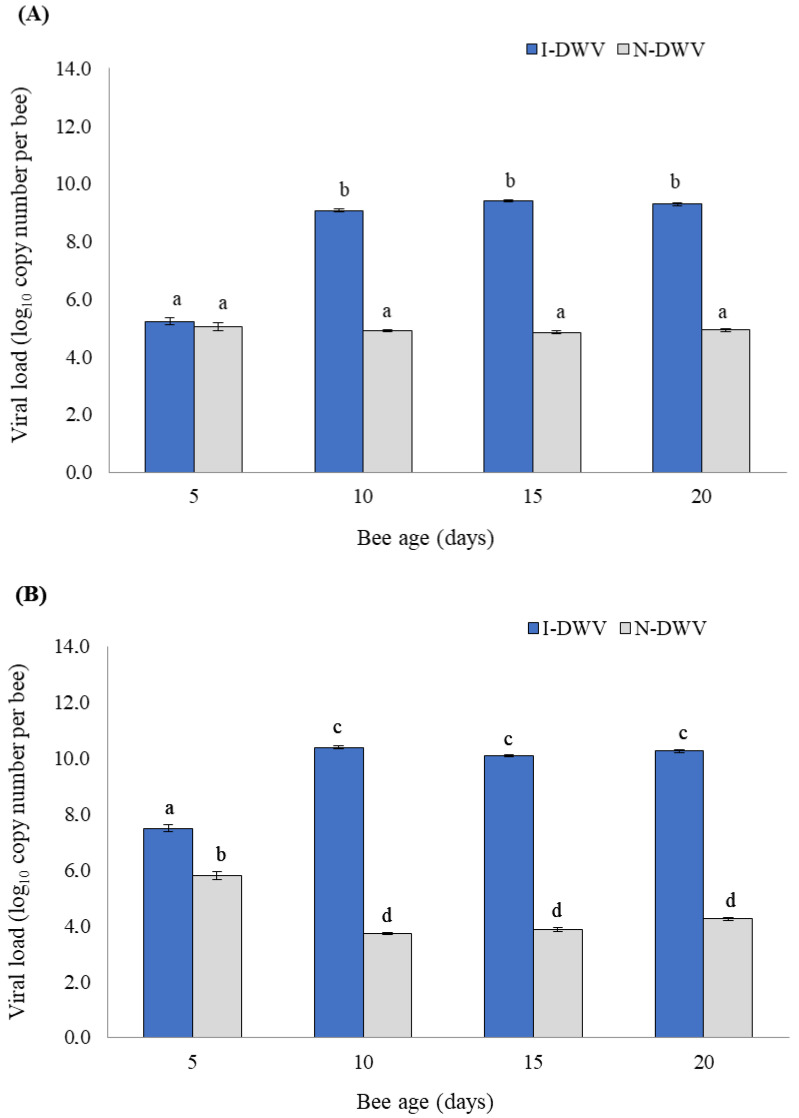
DWV-A load measured in the antennae (**A**) and head (**B**) of *Apis mellifera* of different ages that were inoculated (I-DWV) and non-inoculated (N-DWV). Means (±SE) with different letters indicate significant differences according to the Bonferroni test (*p* < 0.05) (*n* = 216).

The viral load detected in honeybees varied significantly between inoculated (I-DWV) and non-inoculated (N-DWV) bees, but also, these significant changes in viral load were influenced by bee age (Wilks λ = 0.09; F = 157.76; df = 6/414; *p* < 0.001) in both the antenna and head (Figure 1). Viral loads were found to be high and significant (1.0 × 10^9^ viral copy number per bee) in I-DWV bees that were 10- to 20 days old, compared with non-inoculated (N-DWV) bees that also showed a basal viral level (1.0 × 10^4^ to 1.0 × 10^5^ viral copy number per bee) that was not significant (Figure 1A). The viral loads in bee heads also varied among the treatments in bees from 5- to 20 days old; we detected how I-DWV bees showed high viral loads (1.0 × 10^7^ to 1.0 × 10^10^ viral copy number per bee) compared to N-DWV bees (1.0 × 10^4^ to 1.0 × 10^4^ viral copy number per bee) (Figure 1B).

### 3.2. Olfactory Tests

In the Y-tube olfactometer, non-inoculated honeybees (N-DWV) were generally more attracted to the pheromone compound, benzyl alcohol, than worker honeybees that were intentionally inoculated with the DWV-A (I-DWV), except in the youngest bees 5 days after pupal emergence (Table 2). Thus, circa 50 to 60% of N-DWV bees responded significantly to the pheromone compound, measured in 10-, 15- and 20-day-old bees (Table 2). On the other hand, a low proportion (10 to 23%) of the inoculated bees (I-DWV) preferred the pheromone compound. In fact, most non-inoculated bees (N-DWV) showed no preference when tested in the Y-tube olfactometer (Table 2). Also, when the preference between benzyl alcohol or *M. piperita* essential oil was tested in the Y-tube test, significant differences were only observed in 10- and 15-day-old bees, in which case, a significantly higher proportion (almost 50%) of N-DWV bees were attracted to the benzyl In contrast, I-DWV bees of the same age had a low preference (10 to 20%) for the pheromone compound (Table 3). No difference in behavioral preference was observed between N-DWV and I-DWV bees that were 5- and 20 days old when the pheromone and the *M. piperita* essential oil were used as stimuli (Table 3): In fact, 20-day-old non-inoculated bees (N-DWV) significantly preferred the essential oil over the pheromone compound; this response was not found in DWV-A inoculated bees (I-DWV) (Table 3). Furthermore, a significant proportion of I-DWV bees had no preference for benzyl alcohol or the *M. piperita* essential oil (Table 3). 

**Figure 2:**
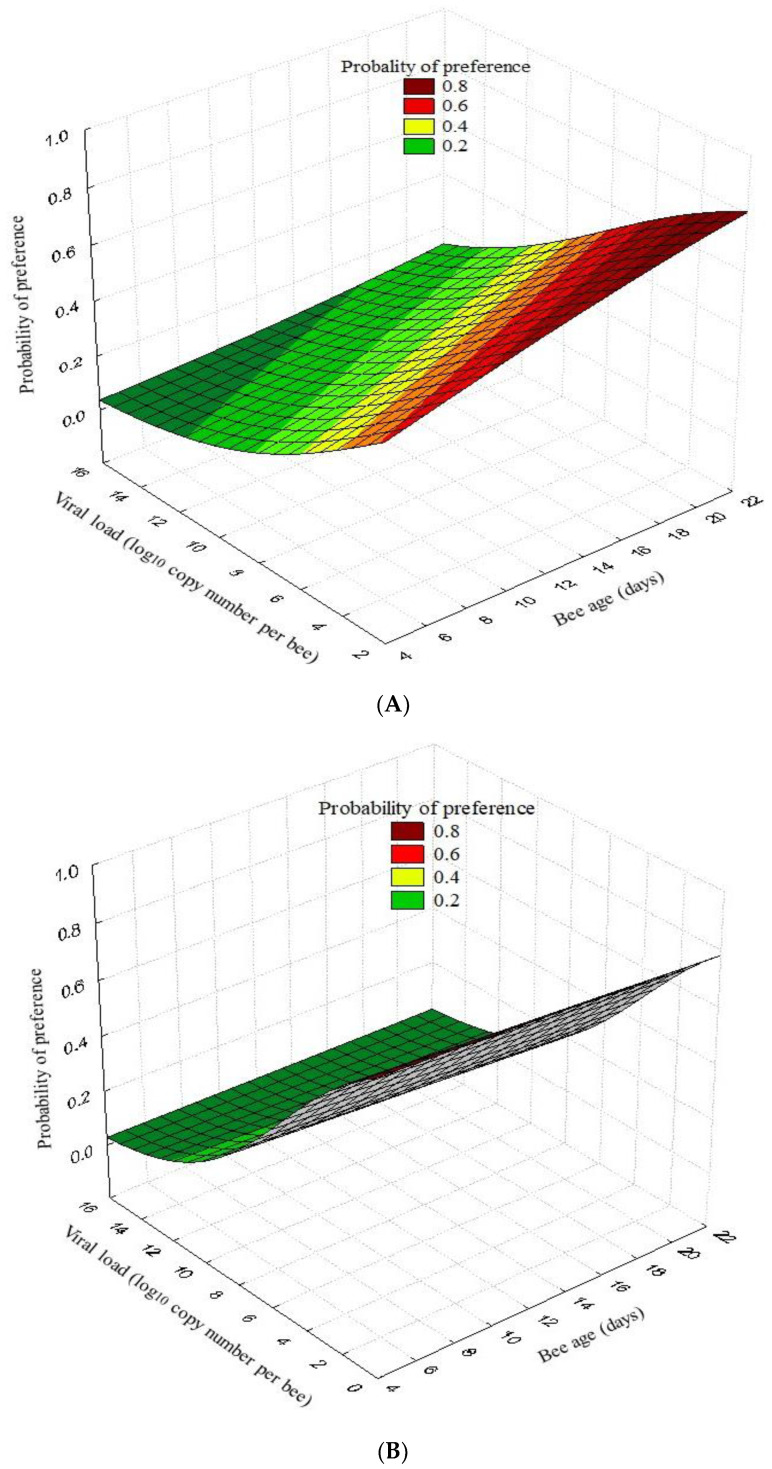
Three-dimensional logistic regression curve representing the probability of the preference of honeybees for a pheromone compound (benzyl alcohol) vs. air (**A**) and a pheromone vs. an essential oil (**B**) in the function of the DWV-A load and bee age in the Y-tube test. When the bees were exposed to the pheromone compound vs. air, the bees that responded by preferring the pheromone were marked as positive (yes = 1, *n* = 76), while those that showed no preference were considered negative responses (no = 0, *n* = 140). When bees were exposed to the pheromone vs. the essential oil, the preference for the pheromone was considered positive (yes = 1, *n* = 63), while bees that showed no preference or preferred another compound were recorded as negative (no = 0, *n* = 153).

The regression logistic analysis showed that the probability of preference for the pheromone compound vs. air via nurse honeybees tested in the Y-tube was significantly influenced by the DWV load (Wald’s χ^2^ = 28.99, *p* < 0.001, Odds ratio = 0.79) and the bee age (Wald’s χ^2^ = 7.47, *p* = 0.006, Odds ratio = 1.08). The probability that nurse honeybees responded to the pheromone compound vs. the essential oil was also significantly influenced by the DWV-load (Wald’s χ^2^ = 27.28, *p* < 0.001, odds ratio = 0.72) but not by the bee age (Wald’s χ^2^ = 0.72, *p* = 0.395, odds ratio = 0.98) (Figure 2B).

### 3.3. Gene Expression

**Figure 3:**
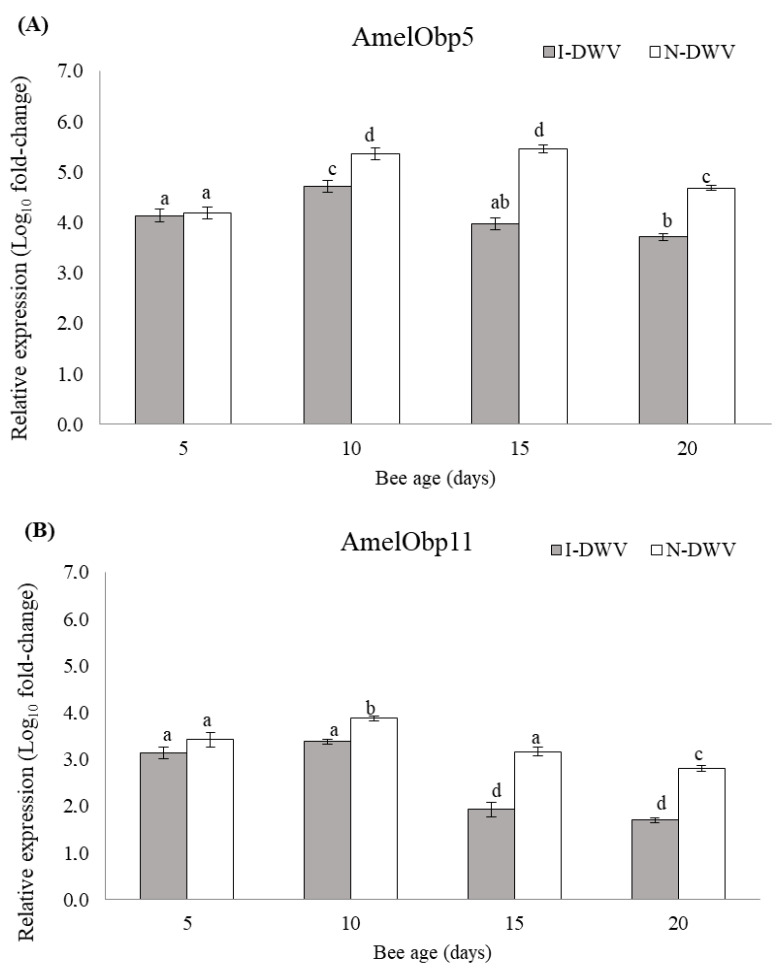
Gene expression of genes coding for OBPs in adult *Apis mellifera* antennae of different ages that were inoculated (I-DWV) and non-inoculated (N-DWV) with DWV-A. Means (±SE) with different letters indicate significant differences according to the Bonferroni test (*p* < 0.05) in (**A**) AmelObp5, (OBP5) and (**B**) AmelObp11 (OBP11) genes with relative expression (*n* = 216).

**Figure 4:**
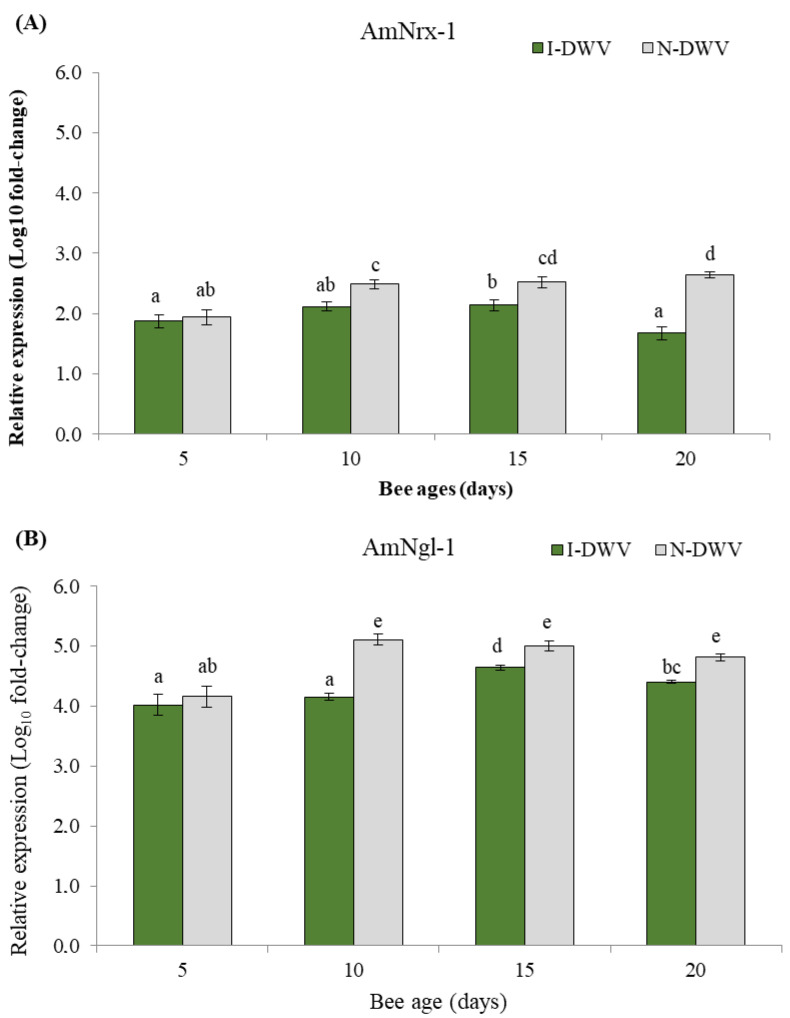
Gene expression of genes coding for neural proteins in adult *Apis mellifera* heads that were inoculated (I-DWV) and non-inoculated (N-DWV) with the DWV-A for different ages. Means (±SE) with different letters indicate significant differences according to the Bonferroni test (*p* < 0.05) in (**A**) AmNrx-1 (Neurexin) and (**B**) AmNgl-1 (Neuroligin) genes with relative expressions (*n* = 216).

Regarding the gene expressions related to OBP proteins and the synaptic neuronal genes in worker honeybees, we detected significant differences in all genes analyzed in bees that were inoculated and non-inoculated with DWV-A. Nevertheless, the viral condition nested in the bee age affected the observed changes in the gene expressions of AmelObp5 and AmelObp11 (MANOVA Wilks λ = 0.48; F = 22.57; df = 8, 414; *p* < 0.001), and also AmNrx-1 and AmNlg-1 (MANOVA Wilks λ = 0.60; F = 14.81; df = 8, 414; *p* < 0.001). We observed in antennae that the genes AmelObp5 and AmelObp11 were significantly down-regulated in inoculated bees (I-DWV) compared to non-inoculated bees (N-DWV) (Figure 3A,B). A significantly lower expression was observed in I-DWV bees from 10- to 20 days old, ranging between 15 and 22% in Amelobp5 and 15% to 65% in Amelobp11 (Figure 3A,B). Similarly, when the synaptic genes AmNrx-1 and AmNlg-1 were analyzed, they were also down-regulated in I-DWV bees (Figure 4A,B). I-DWV bees also showed lower expression levels for both aforementioned genes in the heads of 10- to 20-day-old worker bees. These low expressions ranged from 19 to 53% and 9 to 21% for AmNrx-1 and AmNgl-1, respectively (Figure 4A,B).

## 4. Discussion

It is widely known that olfaction coordinates a large part of insect behaviors, allowing them to select and recognize environmental scents and pheromones [1,2]. Therefore, the ability to respond behaviorally to an odor source greatly depends on the proper functioning of the olfactory system and the different molecules that interact with this system [32]. 

Odorant-binding proteins (OBPs) have been described as essential molecules for scent recognition [33]”. These proteins, after participating in the recognition of environmental compounds, bind to a transmembrane receptor, generating a stimulus in the form of a synapse that is interpreted in the brain; thus, it is understood that these proteins act as the first bridge between the environment and insects [33]. Their role in behavioral responses has been evidenced in *Drosophila melanogaster* Meigen mutants where OBP genes have proven to be responsible for ethanol perception being silenced, resulting in insects that are deficient in this protein, thus showing abnormal behaviors with respect to aroma compared to wild insects [34]. A similar case was described in other insects, such as *Spodoptera litura* F., where the gene coding for the pheromone binding proteins (PBPs) SlitPBP1 and SlitPBP2 was silenced; thus, the insect showed lower behavioral responses. The mutant insects, in this case, were less attracted to the pheromone when used as a stimulus, which was associated with a decrease in the perception and olfactory sensitivity to specific compounds perceived by the PBPs [35]. We previously demonstrated that the presence of the DWV-A at high loads in the antennae generated a reduction in olfactory perception evaluated in EAG assays and a reduction in the expression of the genes Amobp5 and Amobp11 coding for OBP5 and OBP11 proteins [22]. Additionally, it has been studied that these genes are significantly expressed in the antennae of 10- and 15-day-old bees [7,8]. These results have allowed a link to be established between these proteins and nurse bee behavior, in which the insects are dedicated to the task of caring for larvae, considering that the OBP5 protein has a high affinity for compounds of the alarm pheromone of diseased larvae, including phenethyl acetate, phenylethanol, and benzyl alcohol, with benzyl alcohol being the most competitive for the recognition site of the protein [7]. Therefore, the decrease in olfactory preferences observed in our study could be partially explained by the gene knockdown of OBP5 and OBP11 proteins in the antennae, which are responsible for benzyl alcohol recognition. Additionally, honeybees rely on neural processes to perform these complex behaviors, which include foraging, hive coordination, and hygienic behavior [36]. Therefore, the alteration of the normal functioning of neural processes compromises insect behaviors in response to perceived stimuli in the environment [37]. Young honeybees artificially inoculated with DWV have been shown to exhibit learning and memory impairment [37]. In addition, Morfin et al. [24] demonstrated that the co-infection of the ectoparasite *V. destructor* and the deformed wing virus (DWV) decreased the memory and learning ability; they also observed that bees with high viral loads, co-infected with *V. destructor*, decreased the gene expressions associated with the pre- and post-synaptic proteins AmNrx-1 (neurexin) and AmNlg-1 (neuroligin). Thus, they associated a decrease in these genes with a loss and reduction in the learning capacity of bees with high viral loads that are also parasitized with *V. destructor*. Nevertheless, our study demonstrates that the increase in the viral load in the insect brain in the absence of the *V. destructor* mite causes decreases in these pre- and post-synaptic gene expressions. This is one of the few studies to date that evidenced this consequence with only the presence of high viral loads.

Shah et al. [23] reported how the DWV alters the cell structure in critical regions of the brain, including the neuropils responsible for vision and olfaction, and is also capable of dividing different sections of the sensory perception lobes, which could compromise physiological functions provided that this pathogen alters the expression of different genes. Therefore, there is evidence that the DWV affects the capabilities and physiological functioning of sensory organs in bees [21,22,38], which could explain why this alteration in behavioral responses was observed in our study. Furthermore, the logistic regression analysis showed a direct relationship between the viral load detected in the body (Tables S1 and S2, Supplementary Material) and the probability of a preference for specific aromas by young honeybees (Figure 2). This study indicates that a high level of DWV-A in bees reduced the probability that these bees chose the pheromone compound. Nevertheless, a reduction in the behavioral responses of these bees may also be the consequence of multiple altered factors resulting from the presence of the DWV, suggesting that an alteration in the sensory system, specifically a decrease in the sensory perception in the antennae and alterations in the processes of the interpretation of synaptic signals, specifically in the genetic alteration of neuronal genes, could lead these insects to present altered and reduced behaviors. However, their behaviors were not totally deficient because we observed behavioral responses in both treatments in bees that were infected and non-infected with DWV-A. Additionally, behavioral losses have been demonstrated as a consequence of different pathogens of *A. mellifera*, for example, *Nosema* spp. infection causes nursing bees to perform early and poor foraging, altering their feeding preference [39,40].

The decrease in the behavioral response of DWV-A-infected bees could be related to the observed decrease in OBPs and synaptic genes. The decline in the response of healthy 20-day-old bees exposed to the pheromone and essential oil component could be explained, in part, by the age-related distribution of tasks within the hive, known as temporal polyethism [41,42]. The existence of four castes within the hive was proposed in 1986, including cell cleaning (1 to 3 days old), the care and maintenance of the larvae by nursing bees (4 to 12 days old), food processing and nest maintenance by what are known as middle-aged bees (13 to 20 days old) and foraging (in bees more than 21 days old) [43]. Johnson [44] subsequently confirmed the existence of the four castes within the hive, indicating that as the bees mature, their tasks became more limited; thus, 4–12-day-old bees focused solely on brood care tasks, while the 12–20-day-old bees specialized in nectar processing and nest maintenance in preparation for foraging outside the hive. Therefore, it is logical to expect that bees between 15 and 20 days old (in this study) would be more attracted to plant-derived compounds associated with foraging, such as the essential oil of *M. piperita*, than to the pheromone component that was more related to larval scents. In addition, it has been shown that the olfactory perception of foraging bees is greater for pollen-related scents compared to larval-related scents such as the pheromone E-β-ocimene [45]. This could explain, in part, why the behavioral response and preference of inoculated and non-inoculated bees was the same at the end of the trial (20-day-old bees) since non-inoculated bees had a greater preference for the aroma of the plant-derived essential oil over the pheromone compound (Table 3). Therefore, the low preference for the pheromone compound was equal to the preference of the infected bees, and this behavior could be expected given the physiological development of the species itself and the castes within the hive, as described by Johnson [44].

Nevertheless, in bees inoculated with the DWV-A, we did not observe a significant preference for the essential oil in 20-day-old bees. Therefore, we thought that the infection caused by this pathogen could have serious consequences on the physiological development of these insects, affecting the social specialization associated with temporal polytheism, which provides benefits at the group level due to the division of labor that permits increased productivity and reliable task performance. On the other hand, it has been shown that the DWV-A has consequences on the internal cohesion of the hive as it alters the physiological maturation of the nurse bee brain [46], changing the transcriptome towards one similar to that of foraging bees and altering the internal caste by generating premature foragers. This change in caste behavior leads to poor foraging [47], compromising the hive’s integrity.

We demonstrate here that the DWV-A significantly affects young bees (10–15 days old) in the recognition or preference of a specific compound (benzyl alcohol) related to the tasks of nursing honeybees. Therefore, DWV-A-infected nurse bees demonstrated a low perception of the compounds released by the brood that are necessary to maintain their care, which indicates that this brood is poorly cared for, possibly leading to a delay in the development of the colony or its total decline. It is also still unknown whether the DWV affects the preference or attraction for other compounds or pheromones within the colony or aromas present in the flowers that attract foraging honeybees. Therefore, at least two questions remain. Do the DWV-A and/or other DWV variants significantly affect foraging honeybees and their ability to recognize aromas in the field? Can the DWV cause disorientation in the field, leading to a loss of foraging bees because they are unable to locate their colony of origin? Further studies are required to answer these questions. 

## 5. Conclusions

In summary, the high viral load recorded in the antennae and heads decreased the necessary behaviors of nurse bees. The DWV-A also caused a down-regulation of OBPs in the antennae and the synaptic genes neuroxins-1 and neurogilin-1 in bee heads. Therefore, a DWV-A infection could compromise the functioning of the smell and processing of peripheral information, altering the behaviors of nursing bees associated with the aromas of the hive and negatively affecting the survival of bees at the individual and colony levels.

## Figures and Tables

**Table 1:** The primers used in this study.

Primers Names	Sequence	Reference
*AmNrx-1*	F-CTGCTTCGAGCGACGACTAT	[24]
	R-ACGACCGGATGGATGATTGG	
*AmNlg-1*	F-ATGTCGAGGATGCTGCGACTGGA	[24]
	R-ACCTGTGCACTATCTCCTGTTGTA	
DWV-A	F-TATCTTCATTAAAGCCACCTGGAA	[31]
	R-TTCCTCATTAACTGTGTCGTTGAT	
*AmelOBP5*	F-ATGCGGAAATCGTGCTTGCA	[2]
	R-TGCCATTACTCACGGGAAGA	
*AmelOBP1*	F-TGAGGATGTCGAAGCTACGGAA	[2]
	R-CACGGAGCAATAAACGCTATGG	
*β -actin*	F-ATGCCAACACTGTCCTTTCTGG	[31]
	R-GACCCACCAATCCATACGGA	

**Table 2:** The proportion of worker bees that were inoculated (I-DWV) with the deformed wing virus variant A vs. non-inoculated (N-DWV) bees that responded to the pheromone compound benzyl alcohol versus air in the Y-tube test. A chi-square test (*p* < 0.05) was run to detect significant differences in bees that were inoculated and non-inoculated with DWV according to their ages.

Bee Age (Days)	Pheromone (Benzyl Alcohol) vs. Air									
	Status	Pheromone	χ^2^	*p*-Value	Air	χ^2^	*p*-Value	No Preference	χ^2^	*p*-Value
5	I-DWV	0.33	1.36	0.243	0.30	3.75	0.053	0.37	6.70	0.010
	N-DWV	0.20			0.10			0.70		
10	I-DWV	0.10	13.02	<0.001	0.30	3.75	0.053	0.60	3.27	0.071
	N-DWV	0.53			0.10			0.37		
15	I-DWV	0.13	10.80	0.001	0.13	0.00	0.992	0.73	9.64	0.002
	N-DWV	0.53			0.13			0.33		
20	I-DWV	0.23	8.30	0.004	0.23	1.92	0.166	0.53	3.36	0.067
	N-DWV	0.60			0.10			0.30		

The degree of freedom for comparison between the viral status in each bee age for pheromone, air, and no preference response was equal to one (df = *n* − 1).

**Table 3:** A proportion of worker bees that responded to the pheromone compound benzyl alcohol or *Mentha piperita* essential oil in the Y-tube test (A) when worker bees were inoculated (I-DWV) or non-inoculated (N-DWV) with the deformed wing virus variant A. A chi-square test (*p* < 0.05) was run to detect significant differences in bees that were inoculated and non-inoculated with the DWV according to their age.

Bee Age (Days)	Pheromone (Benzyl Alcohol) vs. Essential Oil									
	Status	Pheromone	χ^2^	*p*-Value	Essential Oil	χ^2^	*p*-Value	No Preference	χ^2^	*p*-Value
5	I-DWV	0.23	0.09	0.766	0.07	1.46	0.228	0.70	1.15	0.284
	N-DWV	0.27			0.17			0.57		
10	I-DWV	0.20	7.18	0.007	0.07	1.46	0.228	0.73	11.28	0.001
	N-DWV	0.53			0.17			0.30		
15	I-DWV	0.10	11.43	0.001	0.27	0.32	0.573	0.63	13.61	<0.001
	N-DWV	0.50			0.33			0.17		
20	I-DWV	0.07	1.46	0.228	0.27	11.28	0.001	0.67	17.78	<0.001
	N-DWV	0.17			0.70			0.13		

The degree of freedom for comparison between the viral status in each bee age for pheromone, air, and no preference response was equal to one (df = *n* − 1).

## Data Availability

The data presented in this study are available upon request from the corresponding author. Due to their future use in the development of the team’s own models, the data presented in this study are available upon request.

## Supplementary Materials

The following supporting information can be downloaded at: https://www.mdpi.com/article/10.3390/insects15020080/s1; Table S1: Proportion and load viral (mean) of worker bees that were inoculated (I-DWV) with deformed wing virus variant A vs. non-inoculated (N-DWV) bees that responded to the pheromone compound benzyl alcohol versus air in the Y-tube test; Table S2. Proportion and load viral (mean) of worker bees that responded to the pheromone compound benzyl alcohol or Mentha piperita essential oil in the Y-tube test (A) when worker bees were inoculated (I-DWV) or non-inoculated (N-DWV) with deformed wing virus variant A.
