# Supplementary material for: Nursing Honeybee Behavior and Sensorial-Related Genes Are Altered by Deformed Wing Virus Variant A
Source: Insects. 2024 Jan 23;15(2):80. doi: 10.3390/insects15020080 (PMC10889485; doi:10.3390/insects15020080)
Supplement: Supplementary file 1 [file insects-15-00080-s001.zip › insects-2747104-supplementary.pdf]

Supplementary Material

Table S1. Proportion and load viral (mean) of worker bees that were inoculated (I-DWV) with deformed wing virus variant A vs. non-inoculated (N-DWV) bees that responded to the pheromone compound benzyl alcohol versus air in the Y-tube test

Bee age (days)	Pheromone (benzyl Alcohol) vs Air				
	Status	Load viral	Response pheromone	Response air	No response
5	I-DWV	8.73	0.33	0.30	0.37
	N-DWV	5.48	0.20	0.10	0.70
10	I-DWV	11.94	0.10	0.30	0.60
	N-DWV	4.61	0.53	0.10	0.37
15	I-DWV	12.17	0.13	0.14	0.73
	N-DWV	4.29	0.53	0.14	0.33
20	I-DWV	13.11	0.23	0.24	0.53
	N-DWV	4.14	0.60	0.10	0.30

Table S2. Proportion and load viral (mean) of worker bees that responded to the pheromone compound benzyl alcohol or *Mentha piperita* essential oil in the Y-tube test (A) when worker bees were inoculated (I-DWV) or non-inoculated (N-DWV) with deformed wing virus variant A

Bee age (days)	Pheromone (benzyl alcohol) vs Essential oils				
	Status	Load viral	Response pheromone	Response E.O.	No response
5	I-DWV	8.36	0.23	0.07	0.70
	N-DWV	5.56	0.27	0.16	0.57
10	I-DWV	11.44	0.20	0.07	0.73
	N-DWV	4.79	0.53	0.17	0.30
15	I-DWV	12.30	0.10	0.27	0.63
	N-DWV	4.99	0.50	0.33	0.17
20	I-DWV	58.24	0.07	0.26	0.67
	N-DWV	5.09	0.17	0.70	0.13
